# Beyond One-Size-Fits-All: Precision Mechanical Ventilation in ARDS

**DOI:** 10.3390/jcm15052058

**Published:** 2026-03-08

**Authors:** Saif Azzam, Karis Khattab, Sarah Al Sharie, Lou’i Al-Husinat, Pedro L. Silva, Denise Battaglini, Marcus J Schultz, Patricia R M Rocco

**Affiliations:** 1 Faculty of Medicine, Yarmouk University, Irbid 21163, Jordan; saifazzam2000@gmail.com; 2 Faculty of Medicine, Jordan University of Science and Technology, Irbid 22110, Jordan; kariskhattab@gmail.com; 3 Laboratory of Science and Translation in Critical Illness, Vanderbilt University Medical Center, Nashville, TN 37232, USA; 4 Department of General Surgery and Anesthesia, Faculty of Medicine, Yarmouk University, Irbid 21163, Jordan; 5 Laboratory of Pulmonary Investigation, Carlos Chagas Filho Institute of Biophysics, Federal University of Rio de Janeiro, Rio de Janeiro 21941-971, Brazil; pedroleme@biof.ufrj.br; 6 Department of Surgical Sciences and Integrated Diagnostics, University of Genoa, 16132 Genoa, Italy; battaglini.denise@gmail.com; 7 Anesthesia and Intensive Care, IRCCS Ospedale Policlinico San Martino, 16132 Genova, Italy; 8 Department of Anaesthesia, General Intensive Care and Pain Management, Division of Cardiothoracic and Vascular Anaesthesia & Critical Care Medicine, Medical University of Vienna, 1090 Vienna, Austria; marcus.j.schultz@gmail.com; 9 Department of Anaesthesiology, Rescue- and Pain Medicine, Cantonal Hospital St. Gallen, HOCH Health Ostschweiz, 9007 St. Gallen, Switzerland; 10 Nuffield Department of Medicine, University of Oxford, Oxford OX3 7BN, UK; 11 Mahidol–Oxford Tropical Medicine Research Unit (MORU), Mahidol University, Bangkok 73170, Thailand

**Keywords:** acute respiratory distress syndrome, precision mechanical ventilation, ventilator-induced lung injury, driving pressure, mechanical power, electrical impedance tomography, esophageal pressure monitoring, artificial intelligence

## Abstract

Acute respiratory distress syndrome (ARDS) has traditionally been managed with population-based, protocolized mechanical ventilation strategies designed to limit ventilator-induced lung injury. While these approaches have improved outcomes, they fail to account for the pronounced biological, mechanical, radiological, and temporal heterogeneity that characterizes ARDS. Accumulating evidence shows that patients differ markedly in functional lung size, recruitability, chest wall mechanics, inflammatory burden, and tolerance to ventilatory stress, making uniform ventilatory targets physiologically imprecise and, at times, harmful. This narrative review examines the evolution from conventional lung-protective ventilation toward a precision-based paradigm that aligns ventilatory support with individual patient physiology. We conceptualize ARDS not as a static syndrome but as a dynamic spectrum, viewing the injured lung as a heterogeneous mechanical system susceptible to regionally amplified stress and strain. Within this framework, we discuss key principles underlying precision ventilation, including functional lung size (the “baby lung”), driving pressure, mechanical power, patient–ventilator interaction, spontaneous breathing-associated injury, and the time-dependent evolution of lung mechanics. We synthesize current evidence supporting mechanical, biological, and radiological subphenotyping as complementary strategies to individualize ventilatory management, while critically appraising their current limitations. This review also evaluates bedside tools that may operationalize precision ventilation in clinical practice, including esophageal pressure monitoring, lung ultrasound, and electrical impedance tomography, and examines the role of artificial intelligence as a clinician-directed decision-support aid rather than a prescriptive substitute for physiological reasoning. Implications for clinical trial design, ethical considerations, and future directions toward predictive and adaptive ventilation strategies are also addressed. Precision mechanical ventilation represents a shift from rigid thresholds toward proportional, physiology-guided intervention across the disease trajectory. By integrating evolving lung mechanics, ventilatory load, and patient effort over time, this approach provides a coherent framework for safer and more effective mechanical ventilation in ARDS while preserving the core principles of lung protection.

## 1. Introduction

Acute respiratory distress syndrome (ARDS) has historically been approached as a unified clinical entity, managed with population-based, protocolized ventilation strategies designed to limit overt ventilator-induced lung injury (VILI) [1,2,3]. The introduction of low tidal volume (VT) ventilation and pressure limitation represented a major advance and has undoubtedly improved outcomes. However, it has also become clear that these strategies, while necessary, are insufficient to address the profound heterogeneity that characterizes ARDS.

ARDS is not a single disease but a spectrum of biological, anatomical, and mechanical derangements. Patients differ markedly in inflammatory activity, extent and distribution of lung injury, functional lung size, recruitability, chest wall mechanics, and tolerance to ventilatory load. Moreover, these characteristics evolve over time as the disease progresses or resolves. As a consequence, the mechanical substrate exposed to ventilation is neither uniform across patients nor stable within the same patient, and the relationship between applied ventilatory settings and tissue-level stress is inherently variable [1,2,3].

Despite this complexity, bedside ventilatory management remains largely guided by global indices and population-derived targets, most notably the arterial partial pressure of oxygen (PaO_2_)/fraction of inspired oxygen (FiO_2_) ratio and fixed thresholds for VT and airway pressures [1,3,4]. These variables have value for diagnosis, stratification, and standardization, but they provide little information about how mechanical forces are distributed within the injured lung. Patients with profoundly different functional lung volumes, degrees of regional inhomogeneity, and chest wall loading are therefore frequently exposed to similar ventilatory settings [5]. The consequence is substantial interindividual variability in regional stress, strain, and mechanical energy delivery [6,7], providing a mechanistic explanation for why VILI may persist despite adherence to so-called lung-protective ventilation strategies [8,9].

A more physiologically coherent view conceptualizes the injured lung as a heterogeneous and dynamically evolving mechanical system. In this framework, vulnerability to injury is determined not by airway pressures or VT in isolation, but by the size of the functional “baby lung,” the degree of regional inhomogeneity, the interaction between lung and chest wall mechanics, and the cumulative energy load applied over time. Identical ventilator settings may therefore be either protective or injurious, depending on how and where mechanical forces are distributed within the lung parenchyma [2,3].

These considerations expose a central limitation of contemporary practice: the absence of an integrated physiological framework that translates lung heterogeneity, evolving mechanics, and patient effort into individualized ventilatory decisions at the bedside. Precision mechanical ventilation seeks to fill this gap by shifting the focus from rigid, population-based thresholds toward proportional, physiology-guided support tailored to the patient’s current mechanical and biological state, as shaped by the underlying etiology of lung injury and its temporal evolution.

In this narrative review, we examine the conceptual and physiological foundations of precision ventilation in ARDS. We revisit the injured lung as a dynamic system vulnerable to regionally amplified stress and strain, and discuss key determinants of ventilator-associated injury, including functional lung size, driving pressure (ΔP), mechanical power (MP), patient–ventilator interaction, spontaneous breathing-associated injury, and the temporal evolution of lung mechanics. We synthesize current evidence supporting mechanical, biological, and radiological subphenotyping as a basis for individualized ventilatory strategies and critically appraise both the promise and the limitations of subphenotype-informed care.

We further review emerging bedside tools, including esophageal pressure (Pes) monitoring, lung ultrasound (LUS), and electrical impedance tomography (EIT), and discuss the role of artificial intelligence (AI) as a clinician-directed decision-support adjunct rather than a prescriptive solution. Finally, we explore implications for clinical trial design, ethical considerations, and future directions toward predictive and adaptive ventilation strategies. Together, these concepts position precision mechanical ventilation as a coherent, physiology-driven strategy for safer and more effective respiratory support in ARDS.

## 2. Rethinking ARDS as a Spectrum Rather than a Syndrome

ARDS encompasses a broad and heterogeneous spectrum of lung injury that extends far beyond the simplicity implied by its clinical label [1,10]. Since its original description, it has been recognized as a multifaceted condition arising from diverse insults, including sepsis, pneumonia, and trauma, and expressed across etiological, biological, mechanical, and radiological dimensions. Importantly, these etiological categories are associated with markedly different systemic involvement and prognoses, with substantial variability in mortality despite fulfillment of identical ARDS diagnostic criteria [11]. Distinct biological subphenotypes with different systemic involvement and outcomes have been consistently identified [1,10,12]. From a mechanical perspective, the “baby lung,” defined as the reduced fraction of lung that remains functional and aeratable, highlights that the injured lung is heterogeneous rather than uniform, comprising aerated, poorly aerated, and non-aerated regions and leaving only a limited volume available for ventilation [13]. Imaging studies further show that this loss of aeration is unevenly distributed and evolves over time, reinforcing that both lung structure and function are dynamic rather than fixed [1,10].

Current clinical practice guidelines for ARDS provide robust recommendations for diagnosis, severity stratification, and supportive care, but offer limited direction for individualizing mechanical ventilation beyond population-based targets [4]. These guidelines largely operationalize severity using the PaO_2_/FiO_2_ ratio, derived from the Berlin definition and assessed at a minimum PEEP of 5 cmH_2_O, thereby grouping together patients with markedly different lung volumes, degrees of inhomogeneity, and respiratory system compliance (Crs) [1,12,14]. Uniformity at the ventilator does not translate into uniformity at the tissue level: patients subjected to similar settings experience widely different mechanical stresses and energy loads, which explains why lung injury may persist despite adherence to protective ventilation [1,10,13].

Heterogeneity is not only interindividual but also dynamic within the same patient [15,16]. The size, spatial distribution, and mechanical behavior of the functional lung compartment change with disease progression, inflammatory burden, body position, and applied airway pressures [17]. Accordingly, ventilator settings that are initially appropriate may become injurious over time [18].

Together, these considerations support a shift from viewing ARDS as a homogeneous syndrome toward recognizing it as a dynamic spectrum of injury [19]. In this framework, ventilatory support should be aligned with the patient’s current lung physiology rather than with syndromic severity alone, providing the conceptual basis for precision mechanical ventilation [20,21].

## 3. The Injured Lung as a Mechanical System: Revisiting Pathophysiology

**Figure 1:**
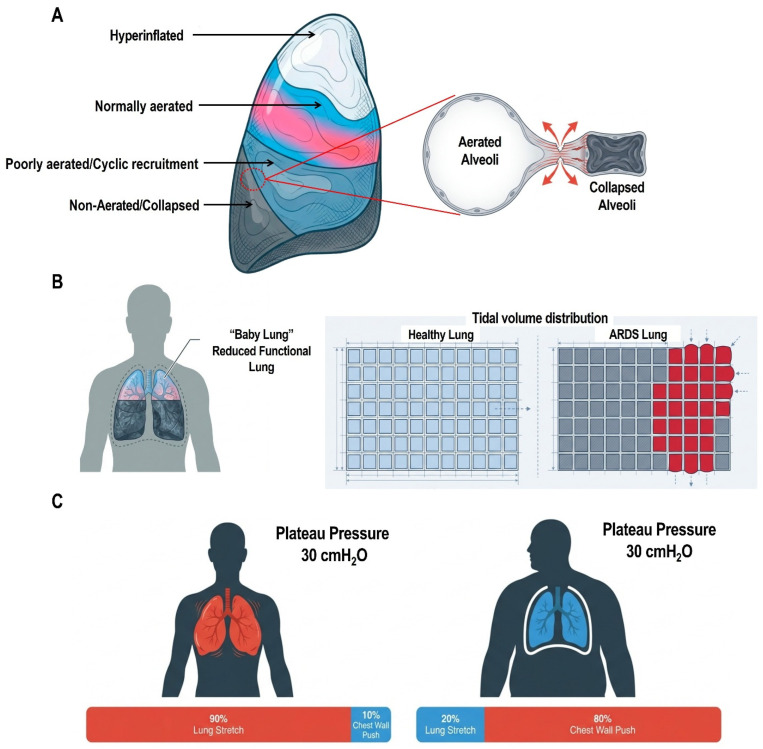
**Mechanical heterogeneity of the ARDS lung and its implications for ventilator-induced lung injury (VILI).** (**A**) The ARDS lung is mechanically heterogeneous, with the coexistence of non-aerated, poorly aerated, normally aerated, and hyperinflated regions. Interfaces between aerated and non-aerated units act as stress raisers, amplifying regional tissue stress despite modest global airway pressures. (**B**) The “baby lung” concept illustrates a marked reduction in functional lung size in ARDS, whereby the entire tidal volume is delivered to a limited ventilatable compartment, resulting in disproportionate regional stress and strain compared with a normal lung exposed to identical ventilatory settings. (**C**) Identical airway plateau pressures may correspond to markedly different transpulmonary pressures depending on chest wall mechanics, highlighting why global airway pressures are imperfect surrogates of true lung-distending stress.

ARDS is fundamentally characterized by mechanical heterogeneity, in which non-aerated or consolidated regions coexist with relatively well-aerated or even hyperinflated areas within the same lung [6,22]. This structural inhomogeneity acts as a potent “stress raiser,” amplifying local stresses at interfaces between regions with different mechanical properties [6,22,23]. As a result, even when global airway pressures remain within conventionally “safe” limits, regional stress and strain may exceed tissue tolerance [6,22,23,24]. The degree of inhomogeneity increases with disease severity and is associated with worse outcomes, helping to explain why global variables such as plateau pressure (Pplat) often fail to reflect the true mechanical burden imposed on the injured lung [6,22,23,24] (Figure 1).

This perspective underlies the “baby lung” concept, which refers to the dynamically reduced fraction of lung parenchyma that remains aerated and available for ventilation in ARDS, rather than to a smaller, structurally normal lung. The size of this functional compartment varies with disease severity, inflammatory burden, and the degree of alveolar recruitment [6,22,23,25]. ARDS is therefore characterized by a mechanically heterogeneous lung in which ventilatory load is distributed over a limited volume, leading to regional amplification of stress and strain. In severe forms of the syndrome, the functional lung volume may be sufficiently small that even modest tidal volumes approach the upper limit of its inspiratory capacity, resulting in disproportionate increases in transpulmonary stress and strain [6,22,23,25]. Under these conditions, Crs serves as a practical surrogate of functional lung size, as it reflects the volume of aerated lung participating in ventilation more accurately than the absolute extent of nonaerated tissue [23,25].

Mechanical power provides an integrative framework linking ventilator settings to VILI [26]. Rather than focusing on isolated variables such as VT or driving pressure (ΔP), MP incorporates VT, ΔP, inspiratory flow, respiratory rate (RR), and PEEP into a unified energy-based construct [26]. From this perspective, VILI can be viewed as the consequence of excessive energy transfer and dissipation within lung tissue over time [22,27]. Importantly, injurious ventilation may therefore occur even at conventionally “protective” VTs if RR or flow is high, increasing the cumulative mechanical energy load applied to a small functional lung [22,25,26].

The lung, however, does not operate in isolation but within the mechanical context of the entire respiratory system, in which the chest wall plays a critical modulatory role [23]. Transpulmonary pressure, the true distending force acting on the lung, depends on the relative elastance of the lung and chest wall. Increased chest wall stiffness, as in obesity or abdominal hypertension, may cause a given airway pressure to generate lower transpulmonary pressure, whereas a highly compliant chest wall may expose the lung to excessive stress for the same airway pressure. Ignoring this interaction exposes the patient to the dual risk of inadequate ventilation and overdistension [22].

Taken together, these concepts emphasize that ARDS is a heterogeneous and dynamically constrained mechanical system rather than a uniform disease [22,24,25]. Variability in lung structure, functional lung volume, energy delivery, and chest wall mechanics limits the effectiveness of “one-size-fits-all” ventilation strategies and supports individualized, physiology-guided ventilatory management [22,23,24,26].

## 4. Subphenotyping for Ventilatory Precision in ARDS

The management of ARDS is increasingly informed by recognition of its marked biological, mechanical, and radiological heterogeneity, moving beyond uniform protocolized ventilation toward a more physiology-oriented framework [28]. Current syndromic definitions rely primarily on hypoxemia and imaging abnormalities and therefore provide limited insight into the mechanisms that govern tolerance to ventilatory stress [12,28]. In this context, subphenotypes can be defined as biologically or physiologically distinct forms of a shared clinical syndrome that arise from different dominant pathobiological or mechanical processes. While conceptually attractive, most evidence supporting this framework derives from observational or post hoc analyses, and prospective validation of subphenotype-guided ventilation remains limited [28].

### 4.1. Mechanical Subphenotypes

Mechanical stratification reflects differences in functional lung volume and pressure–volume behavior of the “baby lung” [28,29]. ΔP, defined as VT divided by Crs, has emerged as a robust indicator of the intensity of mechanical stress applied to the lung. In analyses including more than 3500 patients, each one-standard deviation increase in ΔP (≈7 cmH_2_O) was associated with a 1.4-fold increase in mortality, even when Pplat and VT were within conventionally protective ranges [29,30]. These data indicate that tolerance to ventilatory load varies widely among patients.

A complementary mechanical dimension is lung recruitability, defined as the potential for previously non-aerated lung regions to reopen with positive pressure [28,29,31]. Patients with high recruitability typically exhibit larger non-aerated compartments and may show substantial reductions in non-aerated tissue in response to higher PEEP or recruitment maneuvers, whereas poorly recruitable lungs show minimal response [31]. From a physiological perspective, assessing recruitability is central to balancing the competing risks of atelectrauma and overdistension, although its bedside determination remains imperfect [31].

### 4.2. Biological Subphenotypes

Latent class analysis combining clinical data and plasma biomarkers consistently identified two biological subphenotypes with markedly different prognoses [12]. The hyperinflammatory subphenotype is characterized by elevated plasma concentrations of inflammatory biomarkers, such as interleukin-6 (IL-6) and soluble tumor necrosis factor receptor-1 (sTNFR-1), and is associated with higher rates of shock, metabolic acidosis, and mortality [12]. In contrast, the hypoinflammatory subphenotype shows lower biomarker levels and better clinical outcomes [12]. Across cohorts, 90-day mortality has been approximately 45–50% in hyperinflammatory patients versus about 20% in hypoinflammatory patients.

Beyond prognostic enrichment, post hoc analyses of randomized trials suggest that biological subphenotypes may modify treatment response in ARDS [12,32]. In secondary analyses of the ALVEOLI trial, hyperinflammatory patients appeared to benefit from higher PEEP, whereas hypoinflammatory patients showed no benefit and possible harm. These findings are biologically plausible but remain hypothesis-generating and require prospective confirmation [12,28].

### 4.3. Radiological Subphenotypes

Radiological stratification most commonly distinguishes focal from non-focal (diffuse) patterns of lung injury [28,30]. Focal ARDS is characterized by regionally confined consolidations, whereas non-focal ARDS shows more diffuse involvement [30,33]. Quantitative computed tomography remains the reference standard, but LUS provides a pragmatic bedside alternative with acceptable interobserver agreement [28,31,33].

### 4.4. Etiological Subphenotypes: Direct Versus Indirect ARDS

Etiological classification into direct (pulmonary) and indirect (extrapulmonary) ARDS remains one of the most clinically accessible forms of subphenotyping. Direct ARDS results from primary alveolar insults such as pneumonia or aspiration, whereas indirect ARDS develops secondary to systemic inflammatory processes, most commonly non-pulmonary sepsis. Although conceptually simple, this distinction reflects fundamental differences in the predominant compartment of lung injury [34].

Direct ARDS is primarily characterized by epithelial damage, whereas indirect ARDS more often reflects endothelial dysfunction. In clinical cohorts, direct ARDS has been associated with higher circulating levels of epithelial injury biomarkers, including surfactant protein D, while indirect ARDS demonstrates increased markers of endothelial injury, such as angiopoietin-2 and von Willebrand factor, independent of overall illness severity [35].

These biological differences are accompanied by distinct mechanical and radiological patterns. Direct ARDS more frequently presents with focal consolidation, whereas indirect ARDS typically exhibits a diffuse distribution with greater potential for recruitment. Because etiological classification is readily established at the bedside, it offers a pragmatic approach to anticipating differences in recruitability, response to PEEP, and susceptibility to ventilator-induced lung injury [36].

### 4.5. Subphenotype-Guided Ventilation: Clinical Rationale, Evidence, and Limitations

The core premise of precision ventilation is that mechanical support should be matched to the patient’s dominant mechanical and morphological subphenotype rather than applied uniformly across a heterogeneous syndrome [30]. In focal ARDS, where relatively preserved lung units coexist with localized consolidations, lower PEEP is generally preferred to limit overdistension, moderate VTs may be tolerated, and early prone positioning is particularly effective in improving ventilation–perfusion matching. In contrast, non-focal ARDS, characterized by diffuse instability and greater recruitability, more often benefits from higher PEEP and recruitment maneuvers combined with strict limitation of VT to stabilize vulnerable lung units and reduce cyclic collapse [30].

The clinical relevance and potential danger of this approach were highlighted by the LIVE trial. Although the intention-to-treat analysis showed no overall mortality benefit, post hoc analyses demonstrated that erroneous classification of lung morphology led to a mismatch between regional lung mechanics and the applied ventilatory strategy, with direct clinical consequences. Patients with focal ARDS who were treated with high-PEEP strategies, despite limited recruitability, were exposed to excessive regional stress and strain in already aerated lung units, resulting in mortality rates exceeding 60%, compared with approximately 30% among patients receiving morphology-concordant ventilation. These findings indicate that subphenotyping is not merely descriptive; when it misguides ventilatory loading, it alters the mechanical environment of the lung in a direction that can amplify injury and translate into excess mortality [30].

Despite its strong physiological rationale, routine implementation of subphenotype-guided ventilation remains constrained by major practical limitations. Misclassification is a persistent risk, especially outside expert centers, and subphenotypes may evolve over time as lung injury and systemic inflammation change. Moreover, many biomarkers required for biological stratification are not available in real time, and repeated advanced imaging is often limited by patient instability and logistical constraints. Progress in this field will therefore depend on the development and validation of robust, point-of-care tools, such as simplified LUS-based algorithms and rapid biomarker assays, which allow dynamic, reproducible, and clinically actionable alignment of ventilatory strategy with evolving lung pathophysiology [12,28,33].

## 5. Moving Beyond Tidal Volume: From Scaling by Size to Scaling by Capacity

Scaling VT to predicted body weight provides, at best, a rough surrogate for premorbid lung size. In ARDS, however, the ventilatable lung is functionally reduced, unevenly distributed, and highly variable across patients [37,38,39]. Under these conditions, identical VT values normalized to body size may impose profoundly different mechanical loads on the remaining aerated lung [22,37,39]. Because Crs reflects the size of the aerated, functional lung compartment, it provides a more meaningful physiological basis for interpreting delivered VT than global or nominal volume metrics [37,39]. When Crs is severely reduced, even conventionally “protective” VT can generate excessive strain within the small fraction of lung that remains available for ventilation [37,39].

Beyond technical limitations in measurement, a more fundamental concern is that commonly used surrogates for setting “safe” ventilatory parameters may introduce systematic bias across patient subgroups. Predicted body weight formulas, largely derived from male reference populations, can overestimate functional lung size in women with ARDS. When coupled with sex-related differences in chest wall mechanics and body composition, this miscalibration may result in the delivery of tidal volumes and mechanical power that remain protocol-compliant yet exceed the stress–strain tolerance of the aerated lung. Each subsequent normalization step compounds this initial overestimation, effectively concentrating a given mechanical energy load within a smaller functional lung volume. Unless ventilatory scaling explicitly accounts for sex-specific relationships between body dimensions and lung capacity, population-based protocols will continue to perform adequately for reference groups while systematically exposing others to disproportionate mechanical stress and injury risk [13,40]. These considerations underscore the need to align ventilatory support with functional lung capacity rather than anthropometric surrogates alone.

ΔP formalizes this concept by relating VT to Crs and, therefore, to functional lung size. By expressing volume delivery relative to the lung’s capacity to accommodate it, ΔP provides a closer approximation of the cyclic distending load applied to the ventilated parenchyma [37,38,39]. Consistent clinical and experimental data show that ΔP is more strongly associated with outcome than VT or Pplat considered in isolation, supporting the concept that VILI is governed less by absolute pressure or volume than by the relationship between the two [38,39].

Nevertheless, airway pressures remain imperfect surrogates for tissue-level stress in ARDS. Because the injured lung is structurally inhomogeneous, regional deformation is amplified at interfaces between aerated and non-aerated units. In addition, the transpulmonary stress generated by any given airway pressure depends on the relative contributions of lung and chest wall elastance [22,37,39]. As a result, both Pplat and ΔP may substantially underestimate the stress borne by vulnerable lung regions, allowing injurious deformation to persist despite apparent adherence to global safety thresholds [22,37].

These limitations have prompted a shift from static descriptors toward an energy-based framework [37,38]. MP integrates VT, pressure, flow, PEEP, and RR into a single construct describing the energy transferred to the respiratory system per unit time [22,37]. From this perspective, VILI reflects not only the intensity of each breath but also the cumulative energy load applied to a small and fragile functional lung. Even when VT and pressures appear acceptable, high RRs or excessive flow may drive injurious energy exposure [22,37].

Importantly, MP should be understood as a conceptual framework rather than a validated bedside target. Its principal contribution is to reframe VILI as a problem of energy exposure relative to lung capacity, reinforcing the need to interpret any ventilatory setting in the context of the size, mechanics, and structural vulnerability of the individual lung.

## 6. Precision Ventilation in ARDS: Individualizing PEEP and Alveolar Stability

### Clinical Evidence and Rationales for Individualization

If VILI arises from the interaction between mechanical load and a heterogeneous, unstable lung, then PEEP cannot be prescribed according to oxygenation alone [41,42,43]. The physiological effects of PEEP depend fundamentally on lung morphology, recruitability, and the balance between regional overdistension and collapse. This dependence helps explain why large randomized trials applying higher PEEP strategies indiscriminately have failed to demonstrate consistent survival benefit, and in some cases have increased harm, particularly when combined with aggressive recruitment maneuvers [42,44].

These results instead highlight that the mechanical effects of PEEP are strongly context dependent [41,45]. In some lungs, PEEP stabilizes recruitable units and reduces cyclic opening and closing; in others, it primarily increases stress within already open regions. The same intervention may therefore be protective or injurious depending on the underlying mechanical substrate.

This recognition has motivated the development of physiology-guided approaches to PEEP titration. PEEP–FiO_2_ tables remain widely used because of their simplicity, but they ignore interindividual differences in lung and chest wall mechanics [41,42,43]. EIT offers real-time assessment of regional ventilation distribution and allows identification of the trade-off between dependent collapse and nondependent overdistension [43,45]. In a seminal study, Costa et al. proposed an EIT-based method to estimate recruitable alveolar collapse and overdistension during a decremental PEEP trial, demonstrating good agreement with computed tomography and highlighting the regional nature of lung recruitment and overdistension at the bedside [46]. Esophageal pressure-guided strategies aim to ensure a positive end-expiratory transpulmonary pressure, while other approaches target Pplat or Crs [41,47]. Despite their strong physiological rationale, none of these strategies have yet demonstrated clear superiority in terms of hard clinical outcomes. Importantly, these methods interrogate different aspects of the same mechanical problem and should not be viewed as interchangeable or universally applicable. Their limitations converge on a central issue: lung recruitability and regional vulnerability to stress are not directly or reliably captured by any single global bedside variable.

From a mechanistic standpoint, the purpose of PEEP is to reduce dynamic alveolar instability and limit regional strain amplification in a structurally heterogeneous lung [41]. However, recruitability varies widely among patients, and current bedside tools only approximate this property. As a result, mismatches between lung morphology and ventilatory strategy are not merely ineffective but potentially harmful [45].

The central implication is that no single PEEP titration method can be universally optimal. Progress in precision ventilation is therefore more likely to come from matching specific physiological tools to defined mechanical and morphological subphenotypes, rather than from the search for a single superior algorithm. In this framework, PEEP is not a fixed intervention but a context-sensitive instrument whose value depends on how it interacts with the size, stability, and structural organization of the individual lung, as well as its hemodynamic effects, which may be further modulated by adjunctive strategies such as prone positioning [41,44,45].

## 7. Spontaneous Breathing and Patient–Ventilator Interaction

Once patients with severe ARDS are stabilized with appropriate ventilatory support, including consideration of neuromuscular blockade and prone positioning, spontaneous breathing may be reintroduced to preserve diaphragmatic function, improve hemodynamics, reduce sedation, and enhance patient–ventilator synchrony [48,49,50]. In ARDS, however, these benefits coexist with substantial risks. In a mechanically fragile and heterogeneous lung, patient-generated inspiratory effort may amplify injurious forces, making spontaneous breathing highly context dependent [48,50,51,52].

### 7.1. Patient Self-Inflicted Lung Injury

Patient self-inflicted lung injury (P-SILI) describes lung damage driven primarily by excessive inspiratory effort rather than ventilator-applied pressure alone [51,52]. Although definitive causal proof in humans is limited, physiological mechanisms are well supported. Vigorous diaphragmatic contraction generates large negative pleural pressure swings, markedly increasing transpulmonary pressure and lung stress, even when airway pressures and VTs remain within conventionally protective ranges [50,51,52].

Several interacting mechanisms contribute to P-SILI [50,51,52]. Patient effort adds to ventilator pressure, potentially producing transpulmonary pressures that exceed tissue tolerance. The inhomogeneous ARDS lung promotes early inspiratory intrapulmonary gas redistribution (*pendelluft*), causing regional overdistension before any change in global VT [48,50,51,52]. Strong inspiratory efforts also increase the transvascular pressure gradient, favoring pulmonary edema. These processes may remain largely invisible to airway-based monitoring [23,48,50,52].

### 7.2. Monitoring and Control of Inspiratory Effort

Airway-based indices have important limitations [48,52]. ΔP remains prognostic during passive ventilation but underestimates lung stress during assisted breathing, as airway pressure no longer reflects the total distending force acting on the lung. Measures of respiratory drive and effort derived from airway occlusion maneuvers, including P0.1 and expiratory occlusion pressure (ΔPocc), provide valuable information on neural drive and inspiratory effort, but do not directly quantify regional parenchymal stress and should therefore be interpreted cautiously [23,48,52,53,54].

### 7.3. Neuromuscular Blockade and Mechanical Vulnerability

In the early phase of severe ARDS, when spontaneous inspiratory effort may amplify lung stress and contribute to P-SILI during invasive mechanical ventilation, neuromuscular blockade can be applied to control patient effort [48,49,52]. Early paralysis improved survival and oxygenation in the ACURASYS trial [49], but the subsequent ROSE trial found no mortality benefit when compared with a strategy of lighter sedation and higher PEEP [49]. These discrepancies highlight the limits of uniform strategies in a heterogeneous syndrome [23,55].

Post hoc analyses have suggested that patients with higher respiratory system elastance, reflecting a smaller functional lung and greater stress amplification, may be more likely to benefit from suppression of spontaneous effort. However, these findings are derived from secondary analyses and remain hypothesis-generating. Importantly, the proposed benefit was not consistently accompanied by improvements in key physiological markers such as driving pressure, nor was it supported by robust biomarker or subphenotype differentiation. Sensitivity analyses have further shown that the interaction between neuromuscular blockade and elastance is not fully robust across analytical assumptions. Taken together, these data suggest that while elastance may represent a potential signal for differential treatment response, its role as a reliable selector for neuromuscular blockade has not been definitively established, supporting a cautious, individualized approach rather than routine or elastance-driven paralysis [23,55,56].

### 7.4. A Physiologically Coherent Strategy

A binary view of spontaneous breathing is inappropriate [50,51]. The goal is neither uncontrolled effort nor systematic paralysis but allowing spontaneous breathing when inspiratory effort remains within physiologically tolerable limits and suppressing it when excessive effort generates injurious transpulmonary pressures, high work of breathing, or stress amplification in a reduced functional lung. In practice, sedation, PEEP, and neuromuscular blockade should be guided by the interaction between inspiratory effort, lung size, and mechanical vulnerability [50,51,55].

## 8. Mechanical Power and Energy Load as an Integrative Framework

Lung-protective ventilation has evolved from limiting overt barotrauma to addressing the microscopic forces driving VILI [57,58]. Static limits for VT and Pplat, while foundational, do not capture the dynamic interaction between the ventilator and the heterogeneous functional lung, where small aerated volumes undergo repetitive mechanical loading [57,58,59,60].

**Figure 2:**
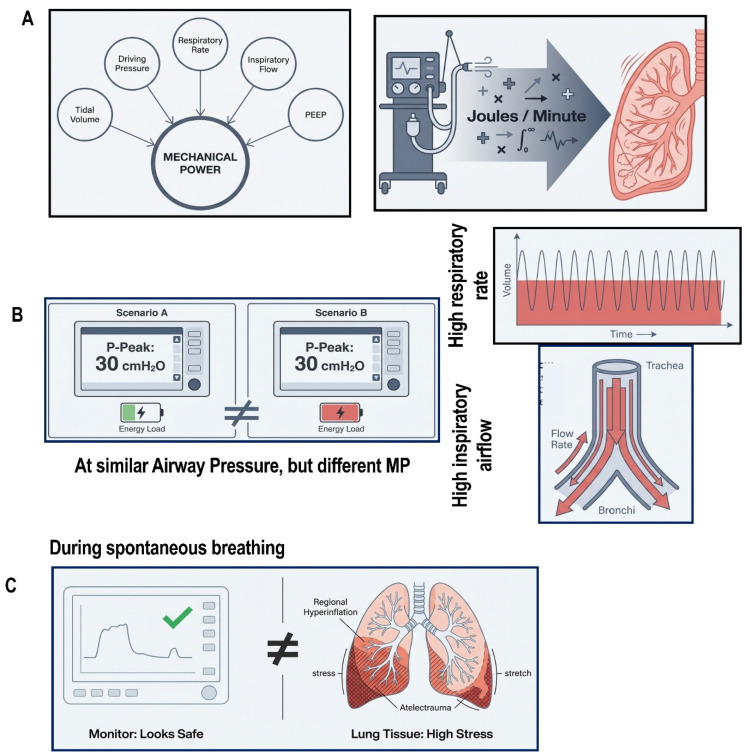
**Mechanical power and patient effort as energy-based determinants of lung stress and injury.** (**A**) Mechanical power represents the total energy transferred from the ventilator to the respiratory system per unit time, integrating tidal volume, driving pressure, respiratory rate, inspiratory flow, and PEEP. (**B**) Different combinations of ventilatory variables may generate similar airway pressures or tidal volumes yet result in markedly different cumulative energy delivery to the lung, illustrating how mechanical power captures injurious load not reflected by static pressure targets alone. (**C**) During spontaneous breathing, patient-generated inspiratory effort adds to ventilator pressure, increasing transpulmonary pressure and regional lung stress even when airway pressures appear “protective,” exemplifying patient self-inflicted lung injury (P-SILI).

In controlled mechanical ventilation, MP extends the stress–strain framework by integrating the cumulative energy delivered over time, reflecting the relationship between ventilator settings and tissue-level stress in the absence of patient-generated effort [59,60,61]. It incorporates VT, ΔP, RR, and inspiratory flow, reflecting both the magnitude and temporal density of energy delivery. This explains why identical volumes or pressures may be tolerated in one patient yet injurious in another, particularly at higher respiratory rates or flow [59,60] (Figure 2).

Observational studies consistently link higher mechanical power to worse outcomes, but absolute values do not uniformly predict injury across patients [59,60,61,62]. In ARDS, normalizing mechanical power to lung compliance or aerated lung volume strengthens its association with outcome, consistent with the principle that a given energy load is more harmful when concentrated within a smaller, stiffer lung [59].

Mechanical power is not yet a standalone bedside target [60,61]. There is no validated threshold, and different ventilator settings can yield identical power values with different biological effects. Within a precision ventilation framework, it should therefore be viewed as an integrative risk signal rather than a control variable. When interpreted alongside lung size and mechanics, it helps identify excessive cumulative energy exposure even when conventional parameters appear acceptable [59,60].

## 9. Time as a Dimension of Precision Ventilation in ARDS

ARDS is a dynamic process in which lung structure and mechanics evolve over time, rather than a static condition defined at a single time point [63,64]. The classical progression from an early, edema-dominated phase toward a later stage characterized by tissue remodeling provides a useful conceptual framework, but in practice, these biological processes frequently overlap and vary markedly among patients [65,66,67]. Precision ventilation therefore requires not only individualization to baseline physiology but also repeated reassessment as lung properties change.

Early in the course of ARDS, a substantial proportion of nonaerated lung often remains potentially recruitable, reflecting the presence of unstable, fluid-filled alveoli rather than irreversible structural collapse [64,65]. However, lung recruitability varies markedly among individuals and is a major determinant of the physiological response to PEEP. When recruitability is high, appropriately titrated PEEP can improve oxygenation and increase Crs by stabilizing previously collapsed lung units [64]. As lung injury progresses and fibroproliferative changes predominate, respiratory system compliance typically declines, and the capacity for further recruitment diminishes [63]. Under these conditions, higher airway pressures are less likely to reopen closed units and more likely to amplify stress within the remaining aerated lung, increasing the risk of regional overdistension [64].

These evolving mechanical conditions imply that ventilatory priorities cannot remain fixed over time [66]. Early management commonly emphasizes prevention of cyclic collapse and excessive strain through low VTs, and PEEP titrated to the individual balance between recruitment and overdistension [64,65,66]. Later in the disease course, when lung volume is often persistently reduced and recruitment potential limited, the focus shifts toward minimizing cumulative mechanical stress while supporting gradual functional recovery [63,66]. Importantly, this temporal shift is not strictly determined by calendar time but by the patient’s evolving mechanical subphenotype.

Precision ventilation therefore requires a dynamic strategy that incorporates timely de-escalation as physiology permits [66]. Persistently high PEEP in lungs with limited recruitability may impair venous return and promote systemic congestion, increasing the risk of extra-pulmonary organ dysfunction [65,67]. Both VT and PEEP must be reassessed repeatedly, guided by changes in lung mechanics and by the lung’s tolerance to mechanical load. Recognizing ARDS as a time-dependent process allows ventilatory goals to evolve from early stabilization toward progressive unloading, rather than remaining anchored to a single protective recipe throughout the course of illness [63,67].

## 10. Integrating Advanced Monitoring into Clinical Decision-Making

Transitioning from protocolized ventilation to true physiological individualization requires tools that can reveal how mechanical forces are distributed within the respiratory system, rather than relying solely on airway pressure and gas exchange [68,69]. Advanced monitoring techniques do not replace clinical judgment but provide essential physiological context for adapting ventilatory settings as lung and chest wall mechanics evolve.

Measurement of Pes allows bedside estimation of pleural pressure and calculation of transpulmonary pressure, offering a direct window into the forces distending the lung [68]. This information can help distinguish whether high airway pressures primarily reflect increased chest wall load or excessive lung stress and can inform PEEP selection in selected patients. However, technical complexity, interpretive uncertainty, and limited availability continue to restrict its routine use in many intensive care units.

Lung ultrasound has emerged as a practical, repeatable tool for assessing changes in lung aeration in response to PEEP by tracking the appearance and resolution of B-lines and consolidations [70]. It is particularly useful for detecting regional recruitment at the bedside. However, because ultrasound cannot reliably identify overdistension, it should not be used in isolation to guide ventilatory pressure settings [70].

EIT extends bedside monitoring by providing continuous, radiation-free imaging of regional ventilation distribution and dynamic changes in lung aeration [69,71]. This technique enables clinicians to visualize the balance between dependent collapse and nondependent overinflation in real time and to adjust ventilatory settings accordingly. Although its physiological appeal is strong, broader clinical adoption will require standardized acquisition protocols, structured training, and clearer evidence linking EIT-guided strategies to patient-centered outcomes [69,71].

Taken together, these tools should be viewed as complementary rather than competing approaches. Their principal value lies in enabling repeated, physiology-driven reassessment of ventilatory strategy as lung mechanics evolve, thereby operationalizing the temporal and individualized approach to ventilation outlined in the preceding sections [47,48].

## 11. Artificial Intelligence and Decision Support: A Tool, Not a Replacement

**Figure 3:**
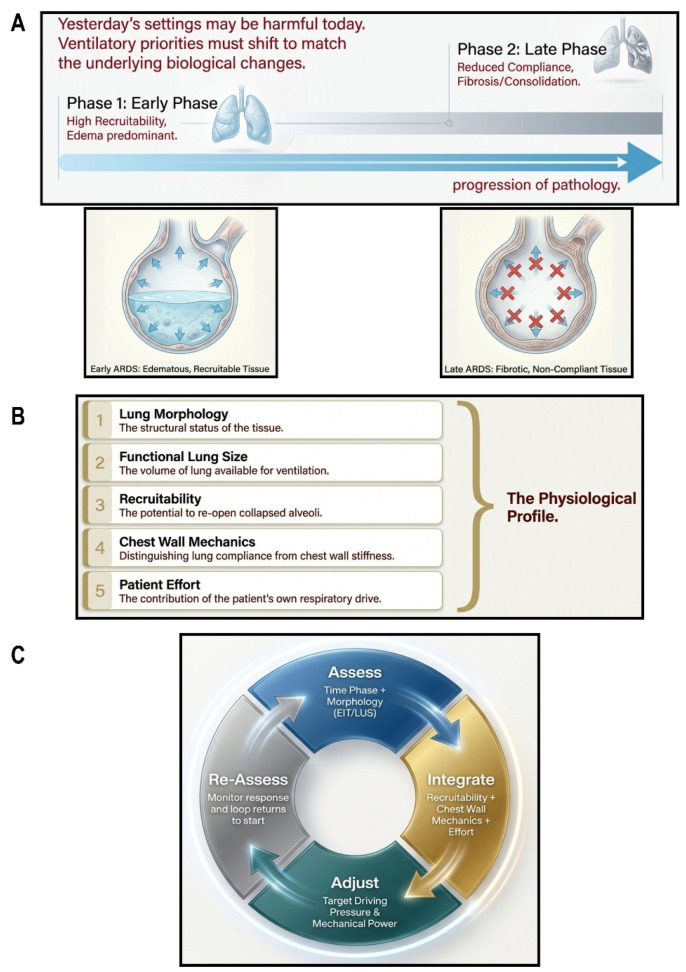
**Precision mechanical ventilation as a time-dependent, physiology-guided strategy in ARDS.** (**A**) ARDS evolves over time from an early, edema-predominant phase with relatively preserved recruitability to later stages characterized by reduced compliance, consolidation, and limited potential for recruitment. As the mechanical substrate changes, ventilatory settings that were initially protective may become injurious if not reassessed. (**B**) Precision ventilation integrates lung morphology, functional lung size (“baby lung”), recruitability, chest wall mechanics, and patient effort to define the individual mechanical context in which tidal volume, airway pressures, and mechanical power are distributed. (**C**) Rather than relying on fixed, population-based thresholds, precision ventilation employs iterative cycles of assessment, integration, adjustment, and reassessment, dynamically adapting tidal volume, PEEP, respiratory rate, driving pressure, and mechanical power to minimize regional stress, strain, and injurious energy delivery as physiology evolves.

The growing complexity of precision mechanical ventilation has markedly increased the cognitive demands placed on clinicians managing ARDS (Figure 3). Modern ventilatory care requires continuous integration of lung mechanics, regional ventilation, gas exchange, hemodynamics, and patient effort, variables that may change over hours rather than days. In this context, artificial intelligence (AI) and machine learning are increasingly explored as decision-support tools, not to replace clinical judgment, but to assist clinicians in navigating high-dimensional bedside data [72].

### 11.1. Machine Learning for Ventilatory Pattern Recognition

One of the most promising applications of machine learning is the recognition of potentially injurious ventilatory patterns that may escape intermittent clinical assessment. By analyzing continuous ventilator waveform data, models can identify combinations of VT, ΔP, RR, inspiratory flow, and patient effort associated with increased risk of lung injury or poor outcomes [73].

This capability is particularly relevant during periods of physiological instability, such as early intubation, during sedation transitions, or when spontaneous breathing emerges. In these settings, harmful mechanical patterns may precede changes in oxygenation or Pplat. Rather than relying on fixed thresholds, data-driven approaches can contextualize variables relative to patient-specific baselines and temporal trends, highlighting trajectories that warrant closer clinical scrutiny [72,73].

### 11.2. Predictive Modeling of Recruitability and Ventilatory Response

Beyond pattern recognition, predictive models have been developed to anticipate physiological responses to ventilatory interventions. Models integrating imaging, respiratory mechanics, and gas exchange data show promise in estimating lung recruitability and predicting responses to PEEP adjustments or recruitment maneuvers [74].

This is clinically relevant because ventilatory “trials” are not benign. Applying high PEEP or aggressive recruitment in poorly recruitable lungs may exacerbate overdistension, impair venous return, and worsen organ dysfunction [75]. Tools that estimate the likelihood of benefit—or risk of harm—could therefore support more informed decision-making. However, given the dynamic nature of ARDS, such predictions must be viewed as probabilistic and used as adjuncts rather than prescriptive directives.

### 11.3. Automation Bias and the Risk of Over-Reliance

The introduction of AI into critical care also carries important risks, particularly automation bias. In high-acuity environments, algorithmic recommendations may be accepted uncritically, especially when workload is high. This risk is amplified in ARDS, where rapid changes in lung mechanics or patient effort can quickly invalidate model predictions if not continuously cross-checked clinically [76].

Moreover, many models are trained on retrospective datasets shaped by historical practices and protocolized care, potentially embedding assumptions that do not generalize across settings or populations [76]. Without rigorous external validation and recalibration, such systems risk reinforcing standardization rather than enabling true physiological personalization.

### 11.4. Embedding AI Within Clinician-Led Precision Ventilation

For AI to meaningfully support precision ventilation, it must operate within a clinician-driven framework emphasizing transparency and physiological reasoning. Rather than issuing commands, systems should highlight risk patterns, suggest candidate strategies, and quantify uncertainty, allowing clinicians to integrate these insights with bedside assessment and imaging [73].

Integration with advanced monitoring—such as EIT, Pes, and LUS—may further anchor AI outputs in real-time physiology. Ultimately, the clinical value of AI will depend less on algorithmic complexity than on how effectively it augments, rather than displaces, expert judgment [72,73].

## 12. Clinical Trials in the Era of Precision Ventilation

Despite the strong physiological rationale, many ARDS ventilation trials have yielded neutral or modest results. While lung-protective ventilation is firmly established, trials of higher PEEP, recruitment maneuvers, or alternative strategies have often failed to show a clear survival benefit [77]. From a precision perspective, this likely reflects indiscriminate application of interventions to heterogeneous populations rather than a true lack of efficacy [78].

### 12.1. Why Traditional Randomized Trials Often Failed

Most trials enrolled patients based on syndromic criteria and hypoxemia severity, without accounting for lung morphology, recruitability, or chest wall mechanics [78,79]. As a result, patients with fundamentally different physiologies received the same intervention. Benefits in one subgroup were often offset by harm in another, producing neutral average effects [79].

Higher-PEEP trials illustrate this problem: patients with high recruitability may benefit, whereas those with low recruitability may experience overdistension and hemodynamic compromise [80,81]. Similar dynamics likely affected trials of recruitment maneuvers, prone positioning outside selected populations, and uniform neuromuscular blockade [82].

Although many physiological and observational studies provide a strong rationale for precision ventilation strategies, randomized clinical trials have frequently yielded neutral results. These findings require careful interpretation and highlight the need for rigorous prospective validation before broad clinical adoption.

### 12.2. Enrichment Strategies and Subphenotype-Specific Trials

Precision ventilation requires enriched trial designs targeting patients most likely to benefit [83]. Enrichment can use mechanical, radiological, or biological markers such as lung morphology, recruitability, or inflammatory profiles [84,85]. Post hoc analyses showing differential treatment responses across subphenotypes support this approach [86]. Although such designs may slow enrollment, they increase interpretability and clinical relevance.

### 12.3. Adaptive Trial Designs for Dynamic Physiology

Because ARDS physiology evolves over time, fixed-design trials poorly capture treatment–time interactions [87]. Adaptive designs, allowing modification of allocation, targets, or eligibility based on interim physiological responses, better reflect clinical reality and permit early discontinuation of harmful strategies in specific subgroups [88].

### 12.4. Meaningful Endpoints Beyond Mortality

Mortality is an insensitive endpoint for interventions aimed primarily at reducing VILI [89]. Precision ventilation also seeks to preserve organ function and shorten ventilation duration [90,91]. Future trials should therefore incorporate mechanistic endpoints, such as ΔP, MP, or ventilation homogeneity, alongside patient-centered outcomes, including ventilator-free days and long-term functional status [92].

## 13. Ethical and Practical Implications of Precision Ventilation

### 13.1. Resource Availability and Global Applicability

Precision mechanical ventilation is constrained by the availability of monitoring tools, staffing, and institutional infrastructure [93]. Many proposed strategies rely on technologies such as EIT, Pes monitoring, or advanced waveform analysis, which are not uniformly accessible across healthcare systems [94,95]. Definitions of precision ventilation that depend exclusively on such tools therefore risk limiting global applicability [90]. Future development of precision ventilation strategies should prioritize simplified, physiology-based approaches that can be applied using standard respiratory mechanics in resource-limited settings. Precision ventilation must be regarded as a scalable concept rather than a technology-dependent model. Although advanced monitoring may enhance physiological assessment, its core principles, aligning ventilatory load with functional lung size, limiting excessive stress and strain, and performing continuous bedside reassessment, remain applicable using conventional respiratory measurements and careful clinical evaluation [77].

### 13.2. Equity in Access to Advanced Monitoring

Unequal access to precision-enabling technologies raises concerns about equity in ARDS care [93]. Well-resourced centers may individualize ventilation using regional monitoring and decision-support systems, whereas others must rely on conventional parameters and clinical judgment [96]. This disparity has implications both for patient outcomes and for the generalizability of evidence derived from precision-focused trials [96].

As precision strategies are progressively incorporated into clinical guidelines and trial design, transparency regarding the feasibility, physiological grounding, and validation of simplified approaches becomes increasingly important.

### 13.3. Training Requirements and Cognitive Workload

Precision ventilation increases cognitive demands by requiring integration of multiple, dynamic physiological signals [93]. Interpreting regional aeration, transpulmonary pressure, or evolving mechanical patterns requires specific expertise, and insufficient training may lead to inconsistent application or misinterpretation [77,97].

These demands extend to the multidisciplinary ICU team. Clear communication of physiological rationale and structured educational strategies will be necessary as precision approaches become more widely adopted [97,98].

### 13.4. Balancing Complexity with Safety

Although individualized ventilation aims to reduce harm, increasing complexity carries inherent risks. Excessive adjustment of ventilator settings in pursuit of theoretical optimization may introduce instability or delay liberation from mechanical ventilation [99].

A balanced approach prioritizes proportional intervention: adapting support when physiological signals suggest clear benefit, while avoiding unnecessary manipulation when the risk–benefit balance is uncertain [100].

## 14. Future Directions from Precision to Predictive Ventilation

The evolution from population-based strategies toward precision ventilation opens the possibility of more predictive management [101]. While current approaches focus on aligning support with measured physiology, future strategies aim to anticipate trajectories of injury and recovery, enabling earlier intervention and safer de-escalation [91].

Closed-loop ventilation systems represent one potential extension of this concept, allowing automated adjustment of settings in response to physiological inputs [102]. However, the heterogeneity and instability of ARDS limit the suitability of fully autonomous control. Development is therefore more likely to favor hybrid systems that assist with routine adjustments while preserving clinician oversight for high-risk decisions [73].

Integration of mechanical, imaging, biological, and clinical data may improve early identification of patients at risk for VILI or delayed recovery [103]. Predictive frameworks capable of tracking changes in ventilatory load, recruitability, or inspiratory effort could support earlier modification or de-escalation of support, provided outputs remain physiologically interpretable and clinically actionable [103,104].

Future strategies may increasingly emphasize early mechanical personalization to limit cumulative lung injury before irreversible damage occurs [105]. Early assessment of lung mechanics and careful titration of ventilatory load at the onset of respiratory failure remain promising but unproven directions [104].

As predictive approaches mature, static thresholds for VT and airway pressure may be complemented by dynamic targets incorporating functional lung size and ventilatory load [17]. Any redefinition of lung-protective ventilation must proceed cautiously and preserve established safety principles across diverse clinical settings [18].

## 15. Conclusions

Mechanical ventilation in ARDS has traditionally relied on population-based rules designed to limit overt injury. Although these strategies have improved outcomes, they do not account for the profound heterogeneity in lung mechanics, recruitability, chest wall properties, and disease evolution that characterizes ARDS. Increasing evidence indicates that VILI is determined not by isolated variables, but by how mechanical forces are distributed within a dynamically changing and mechanically heterogeneous lung.

Precision mechanical ventilation reframes lung protection as a patient-specific and time-dependent process. By integrating functional lung size, ventilatory load, regional aeration, patient effort, and temporal evolution, this approach shifts the focus from fixed thresholds toward individualized physiological interpretation. Importantly, precision ventilation is defined not by any single tool, but by a commitment to aligning support with the mechanical and biological state of the lung.

The limited success of many ventilation trials underscores the consequences of applying uniform interventions to heterogeneous populations. Future progress will depend on trial designs incorporating physiological subphenotyping, adaptive strategies, and endpoints that better reflect lung protection rather than mortality alone. Advances in monitoring, data integration, and decision support may facilitate this transition, provided they remain transparent, interpretable, and clinician-directed.

Ultimately, precision ventilation represents a conceptual shift rather than a prescriptive protocol. Its aim is not maximal individualization, but proportional intervention guided by evolving physiology. By moving beyond one-size-fits-all approaches and embracing patient-specific mechanical reasoning, precision ventilation offers a coherent framework for safer and more effective respiratory support in ARDS.

## Data Availability

No new data were created or analyzed in this study.
